# Supplementary material for: Identification of a DNA Methylation Episignature in the 22q11.2 Deletion Syndrome
Source: Int J Mol Sci. 2021 Aug 10;22(16):8611. doi: 10.3390/ijms22168611 (PMC8395258; doi:10.3390/ijms22168611)
Supplement: Supplementary file 1 [file ijms-22-08611-s001.zip › ijms-1311593-sup/Supplementary_Figures_1_2.pdf]

A

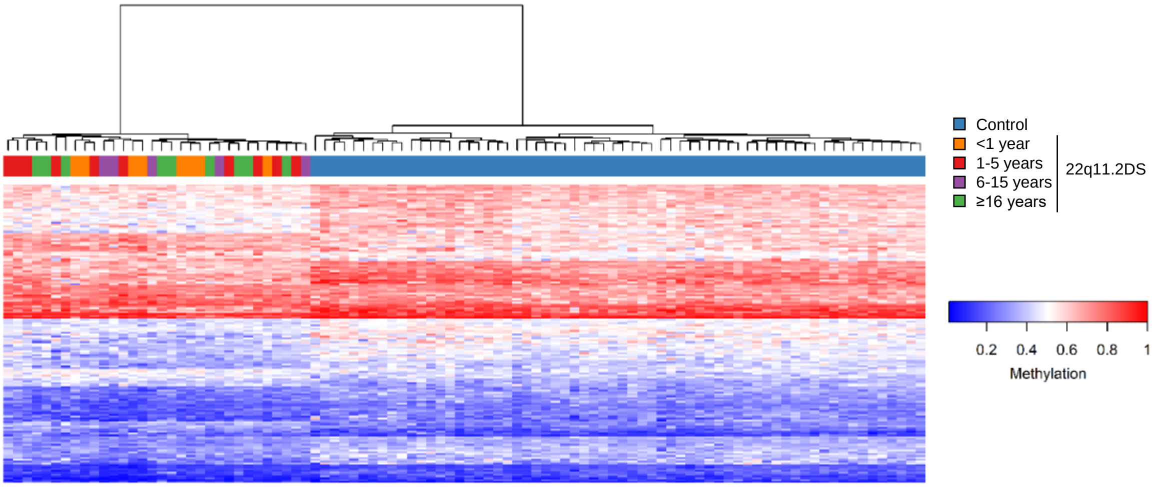

B

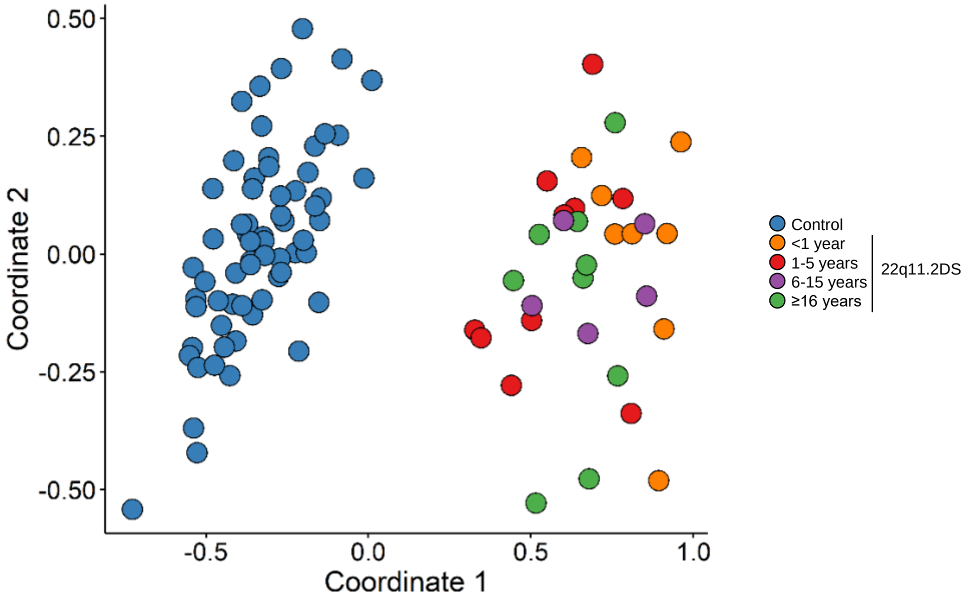

Supplementary Figure 1: Unsupervised clustering of 22q11.2DS and control samples coloured be age. **A.** Hierarchical clustering. Each row represents one microarray probe each column represents one sample. **B.** Multidimensional scaling.

A

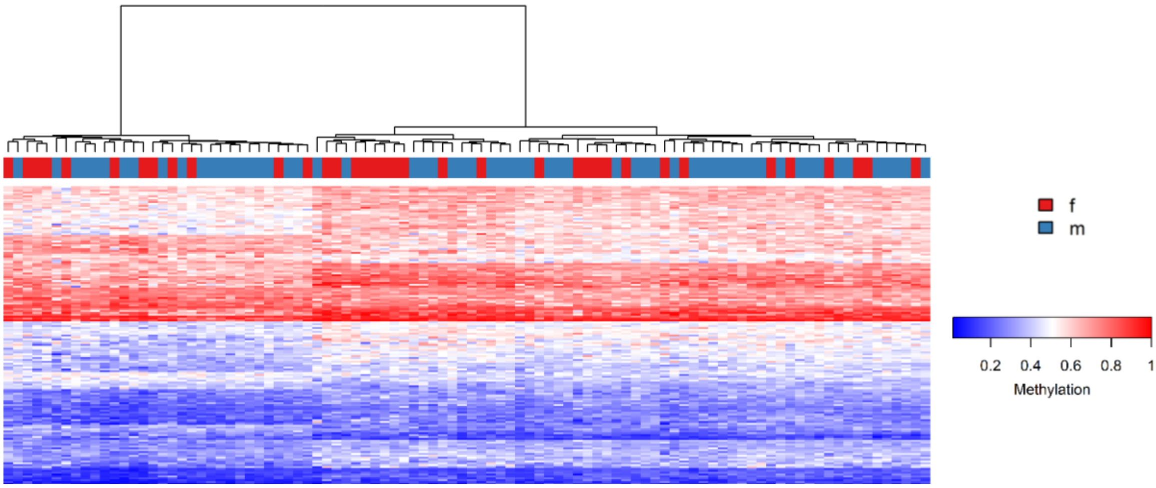

B

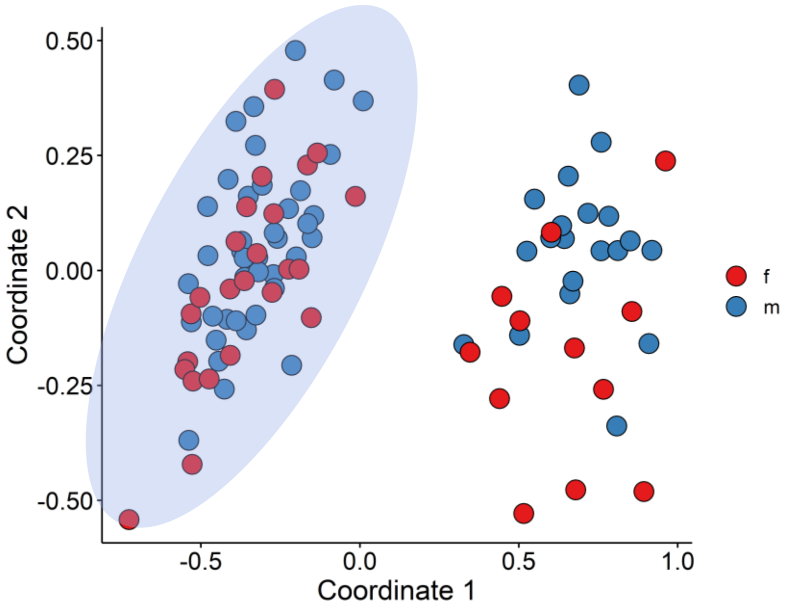

Supplementary Figure 2: Unsupervised clustering of 22q11.2DS and control samples coloured be sex. **A.** Hierarchical clustering. Each row represents one microarray probe each column represents one sample. **B.** Multidimensional scaling, controls encircled in blue.
