# Supplementary material for: Identification of a DNA Methylation Episignature in the 22q11.2 Deletion Syndrome
Source: Int J Mol Sci. 2021 Aug 10;22(16):8611. doi: 10.3390/ijms22168611 (PMC8395258; doi:10.3390/ijms22168611)

Supplementary Figure 3: Leave-1-out cross validation results

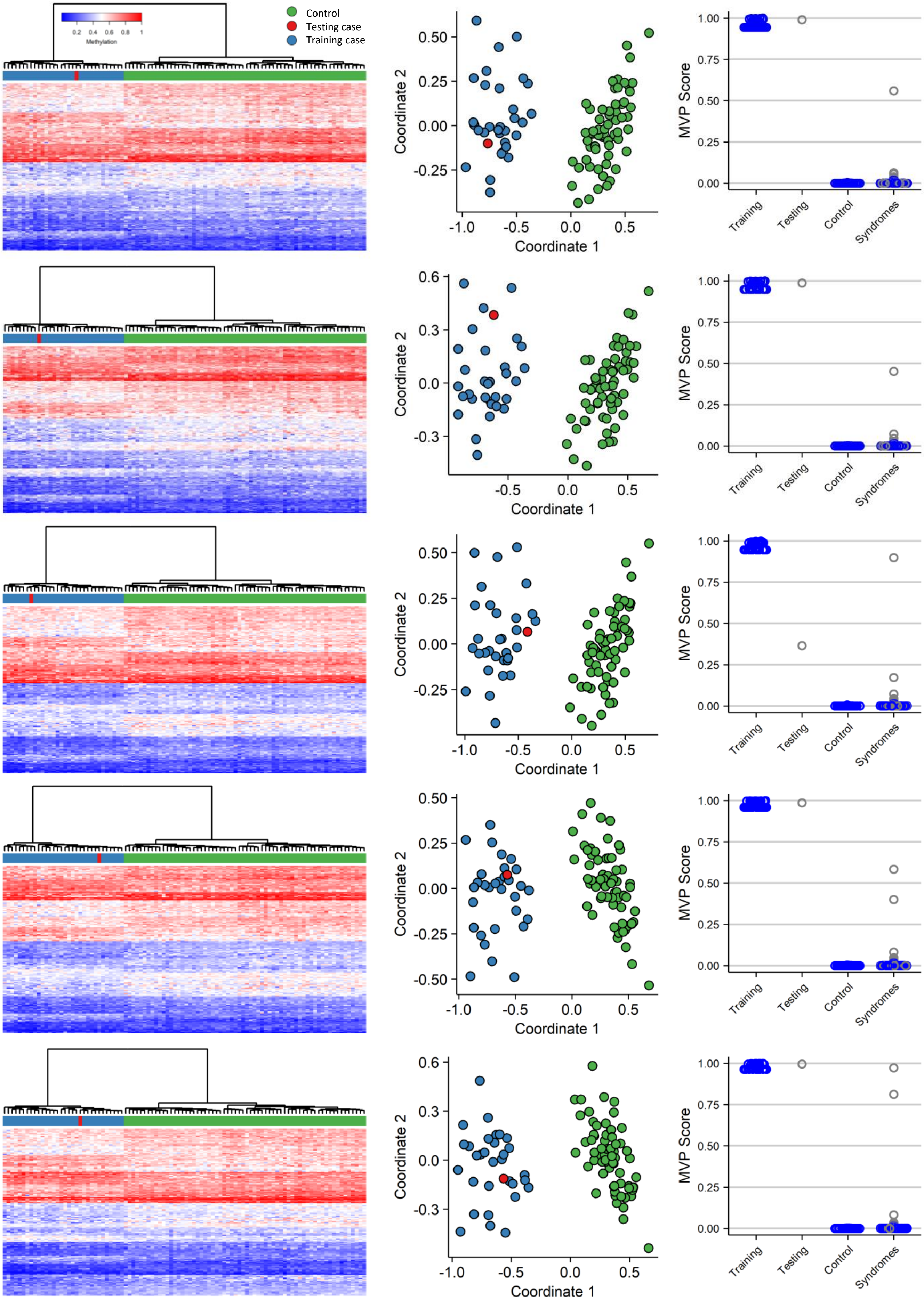

Supplementary Figure 3: Leave-1-out cross validation results

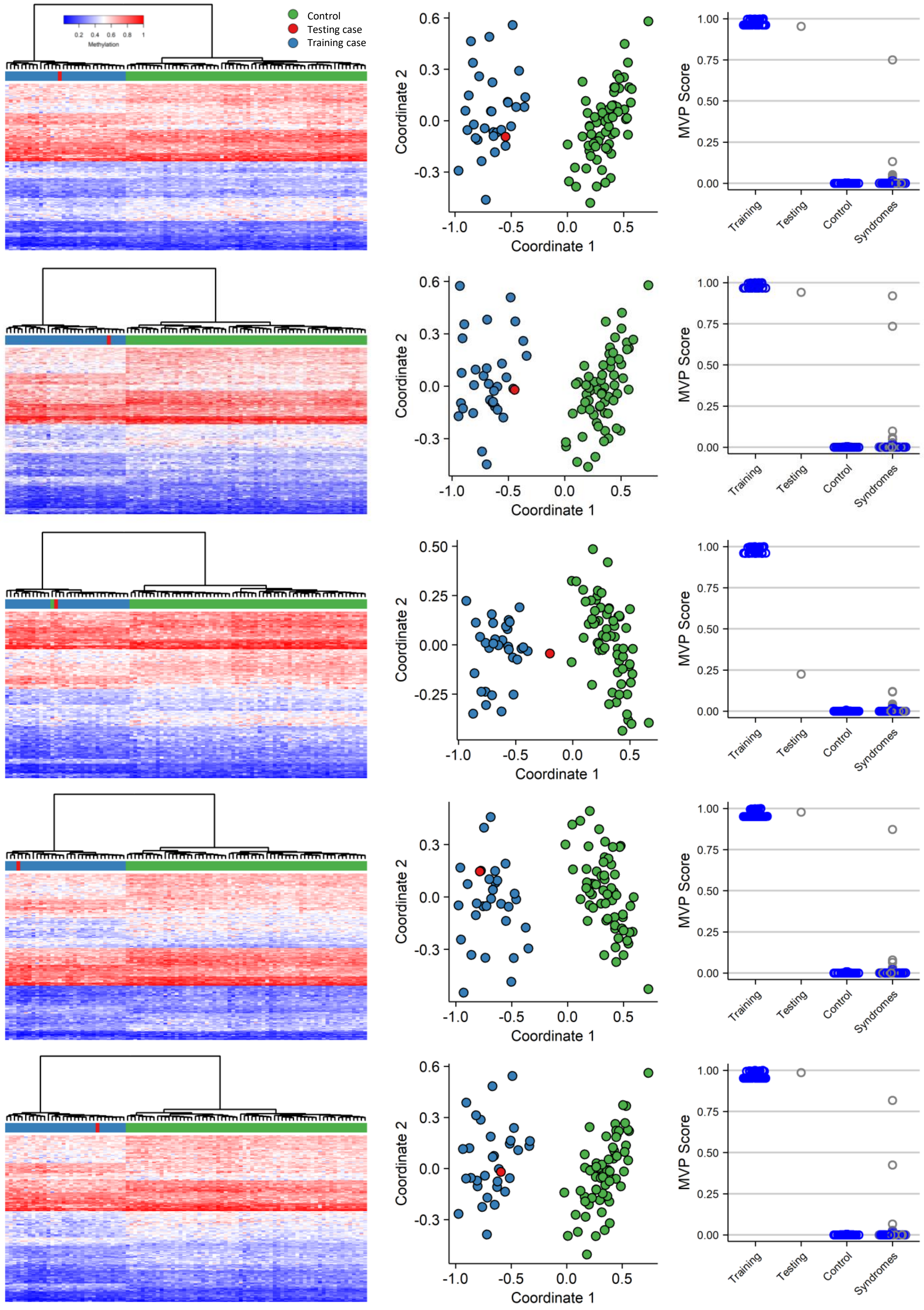

Supplementary Figure 3: Leave-1-out cross validation results

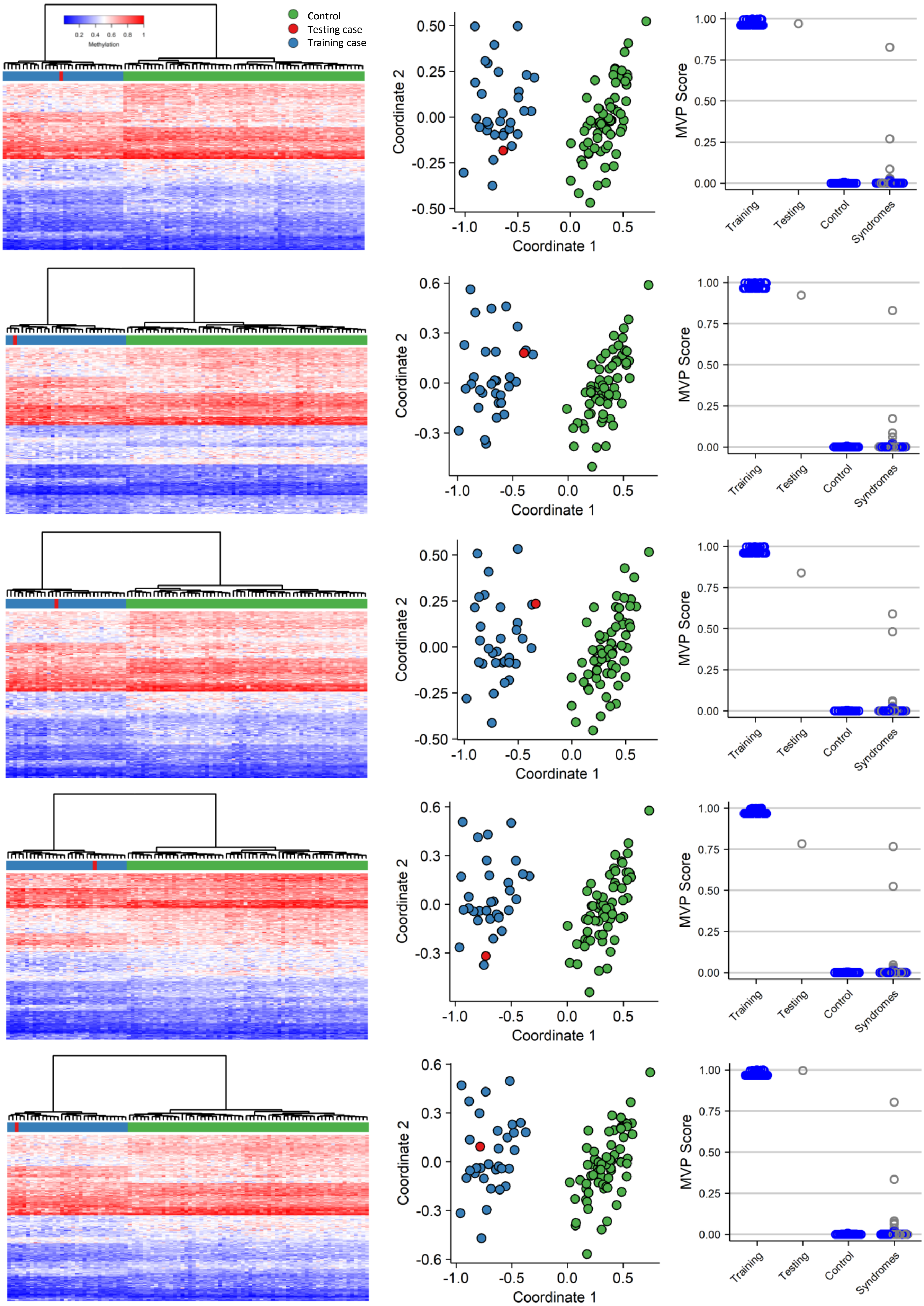

Supplementary Figure 3: Leave-1-out cross validation results

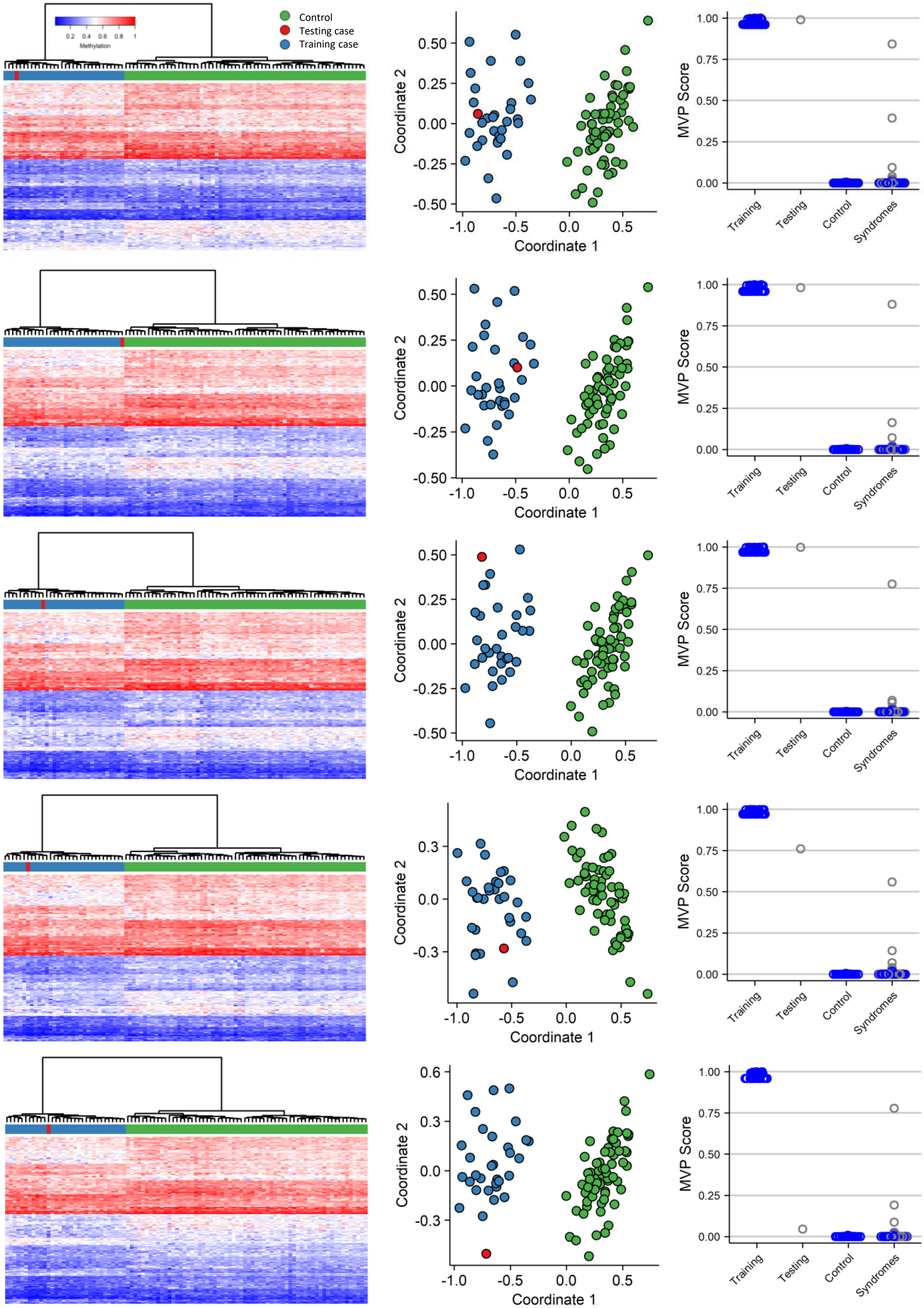

Supplementary Figure 3: Leave-1-out cross validation results

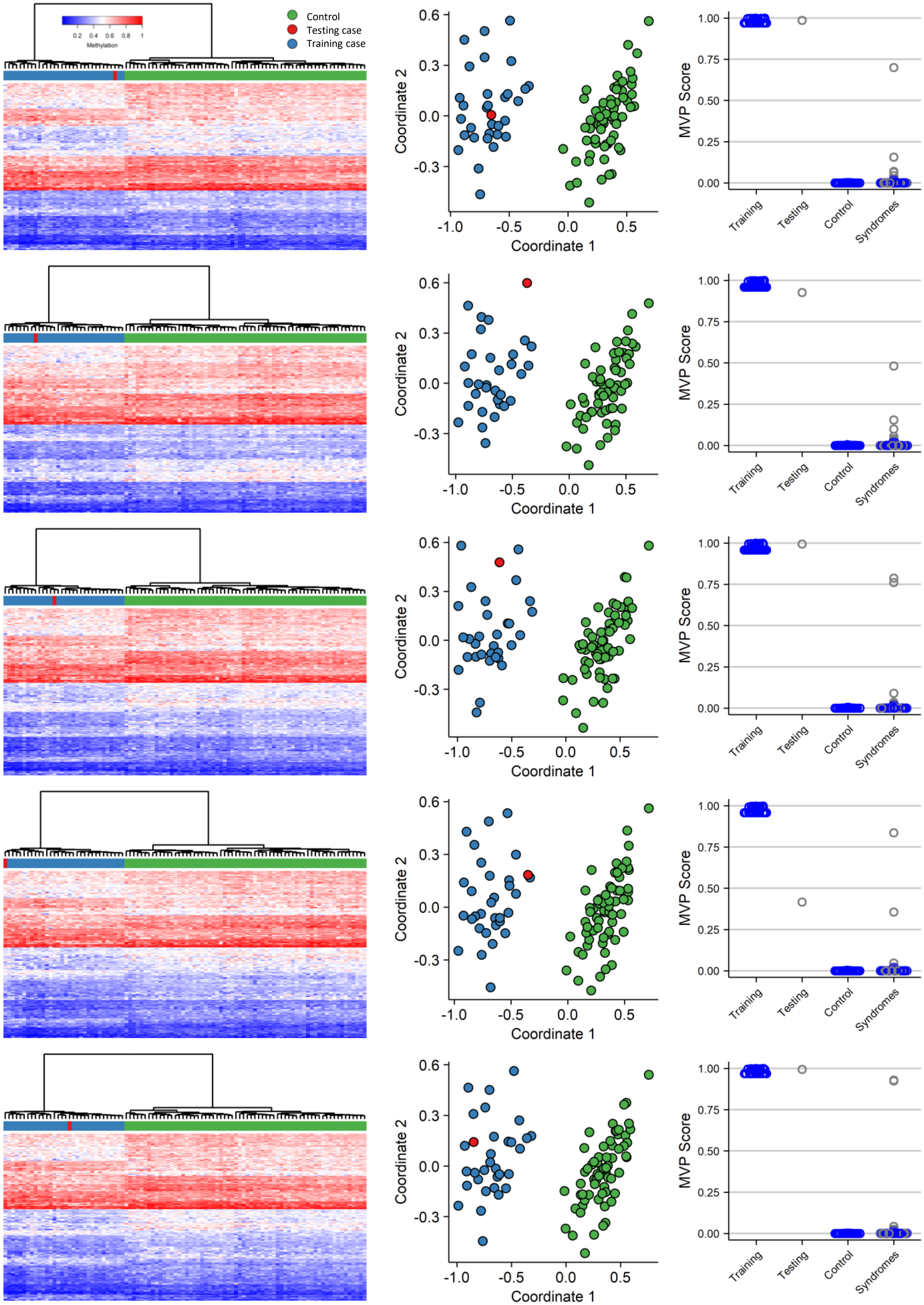

Supplementary Figure 3: Leave-1-out cross validation results

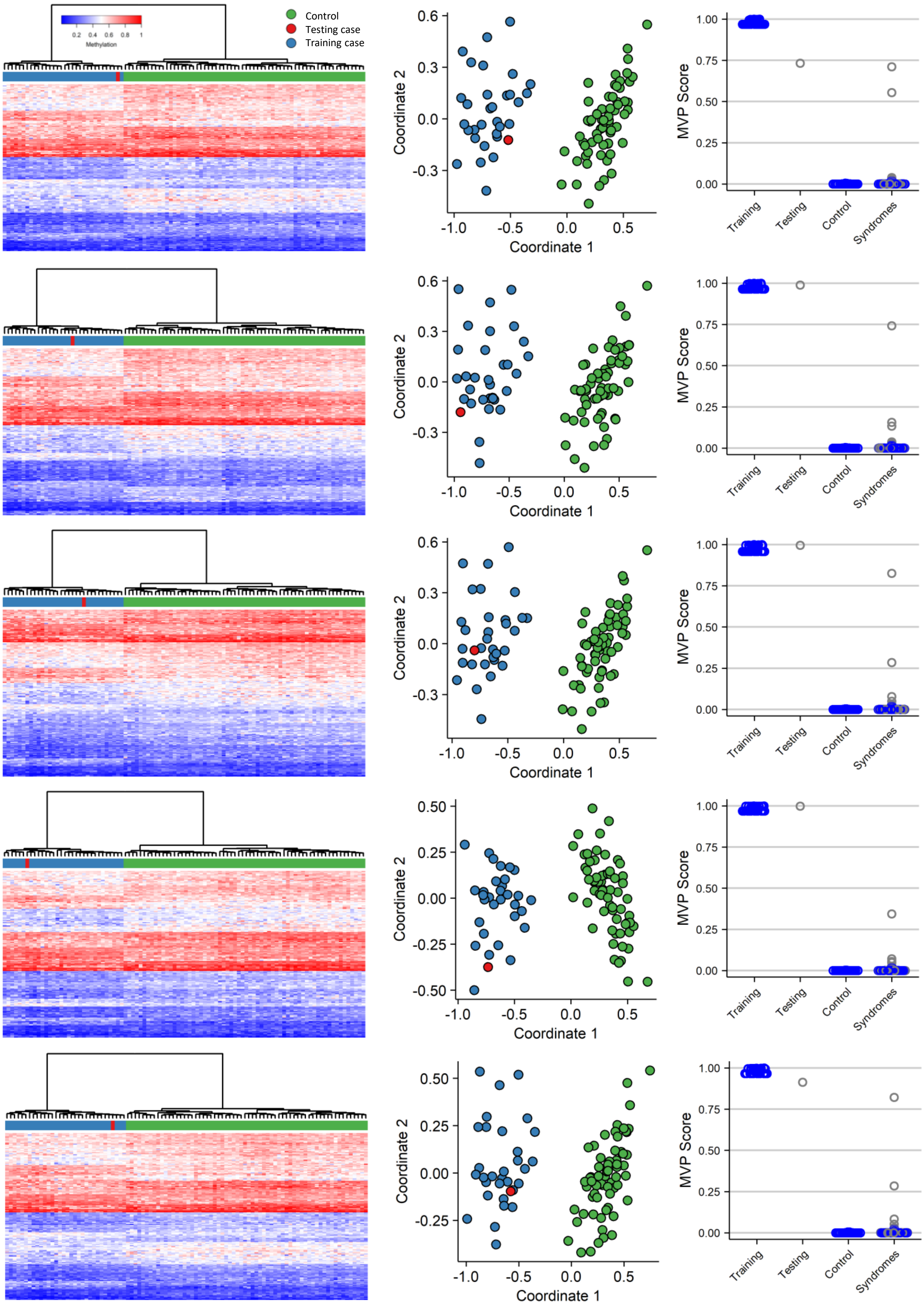

Supplementary Figure 3: Leave-1-out cross validation results

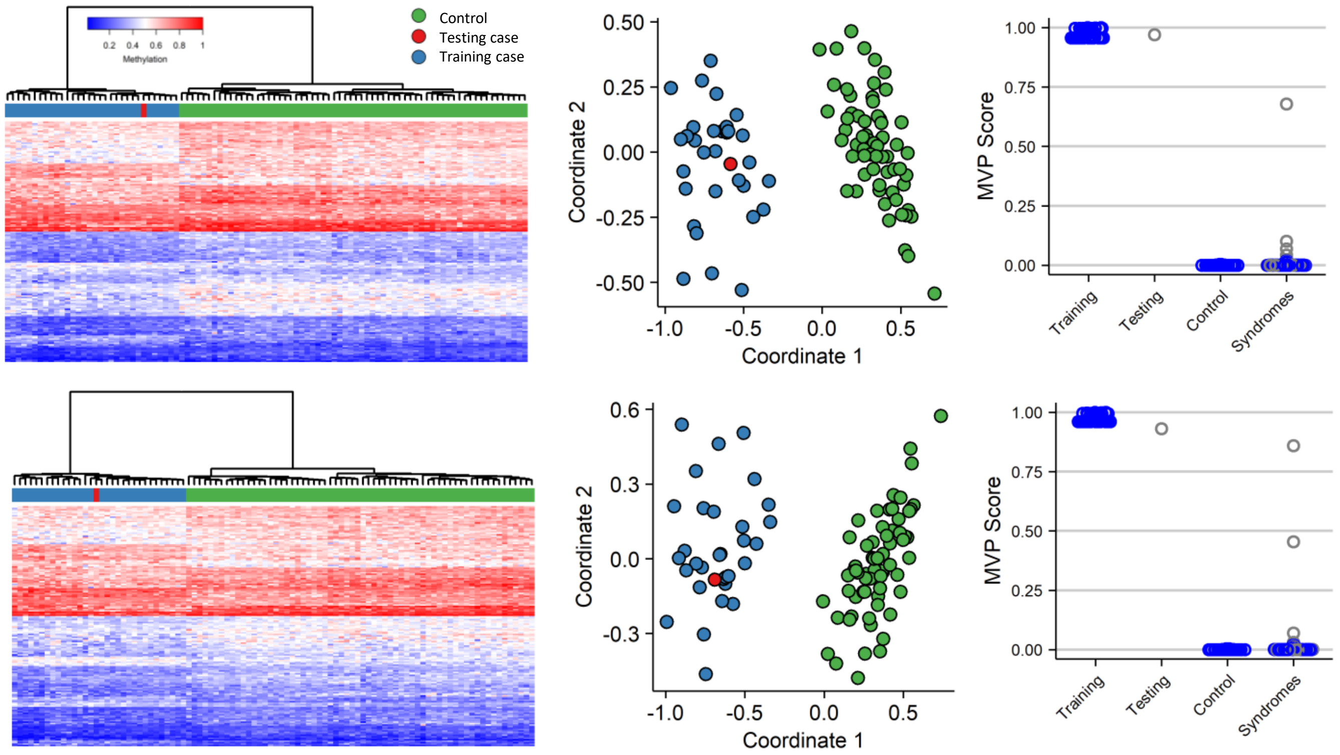

Supplementary Figure 4: Leave-25%-out cross validation results

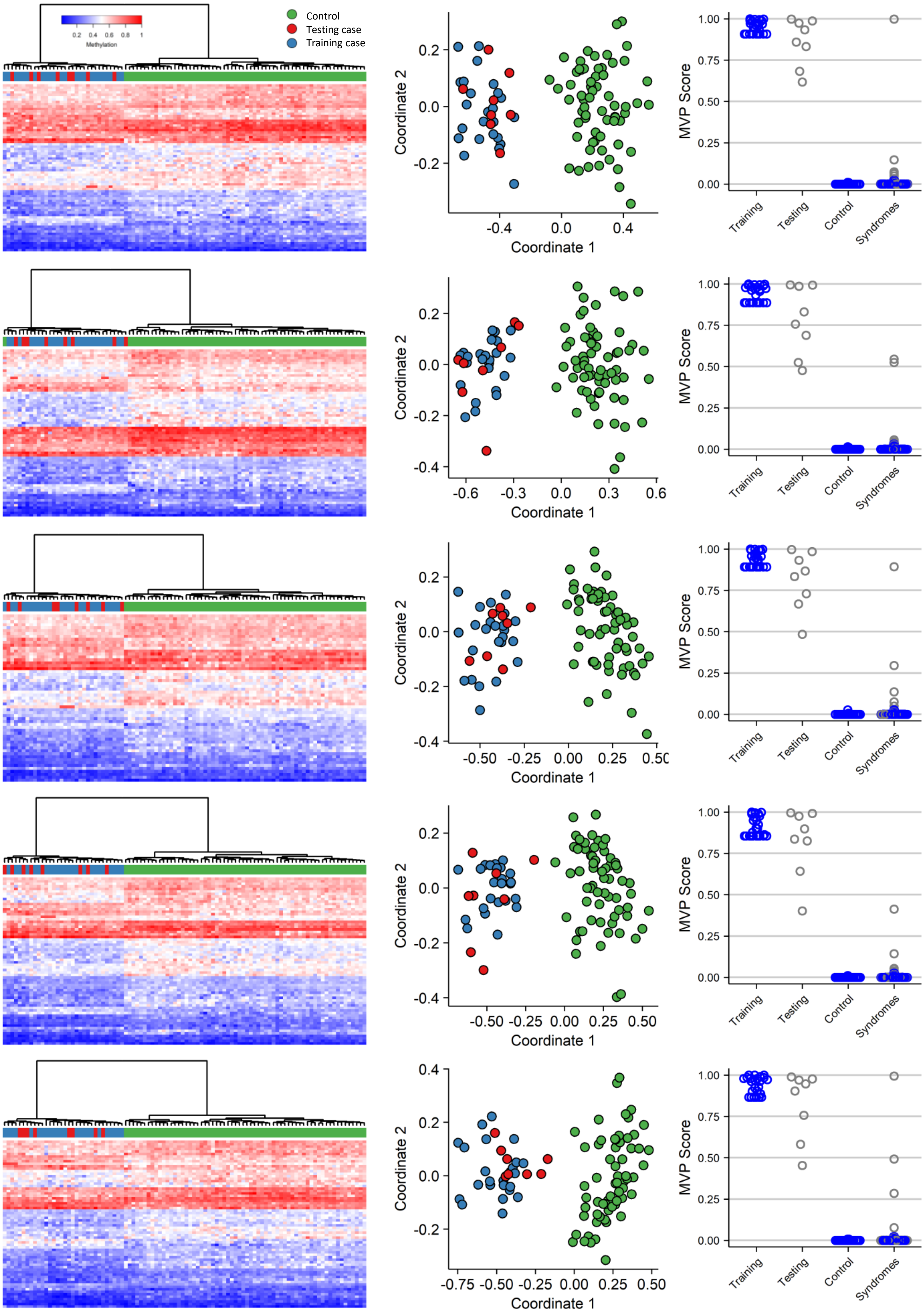

Supplementary Figure 4: Leave-25%-out cross validation results

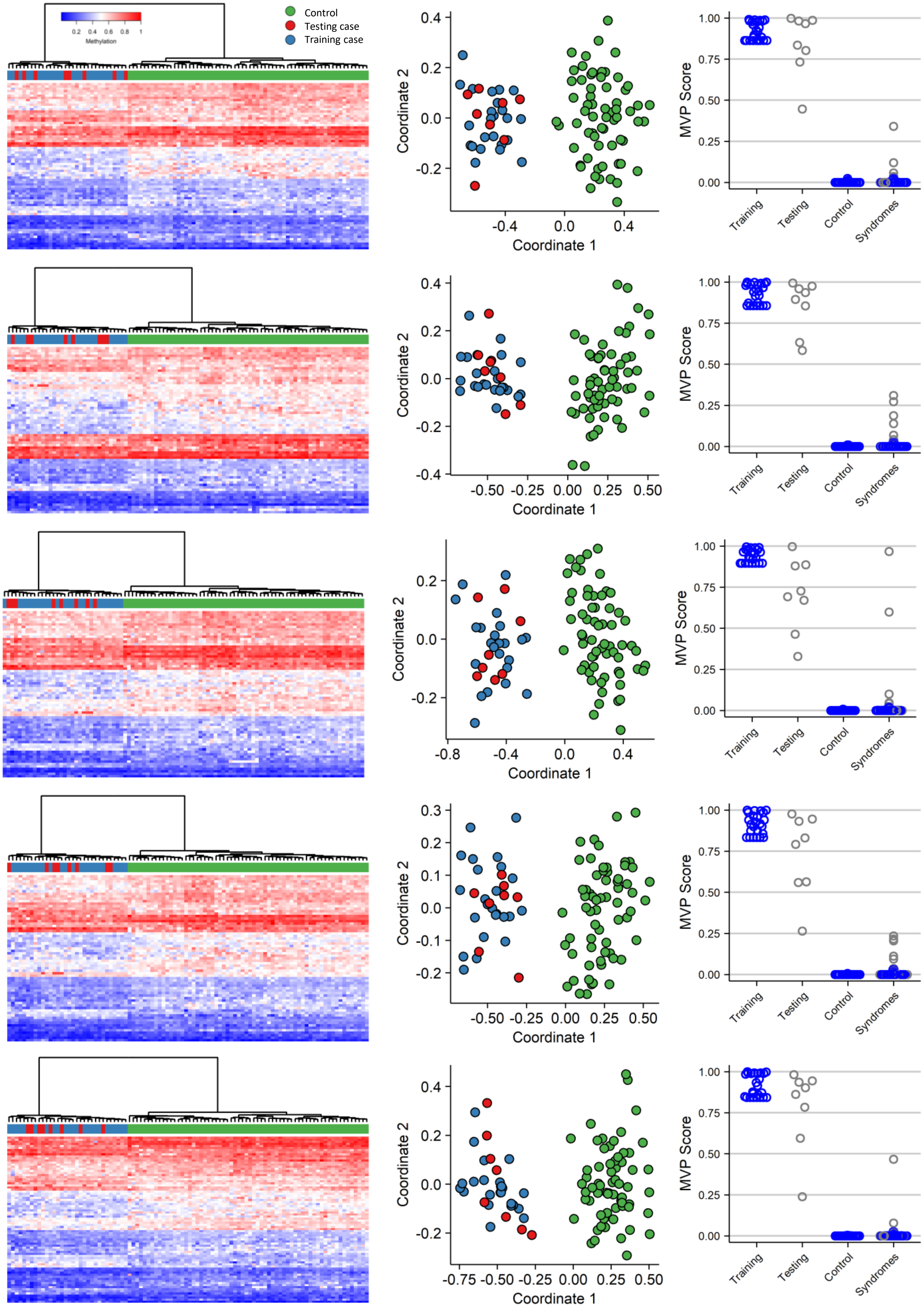

Supplementary Figure 4: Leave-25%-out cross validation results

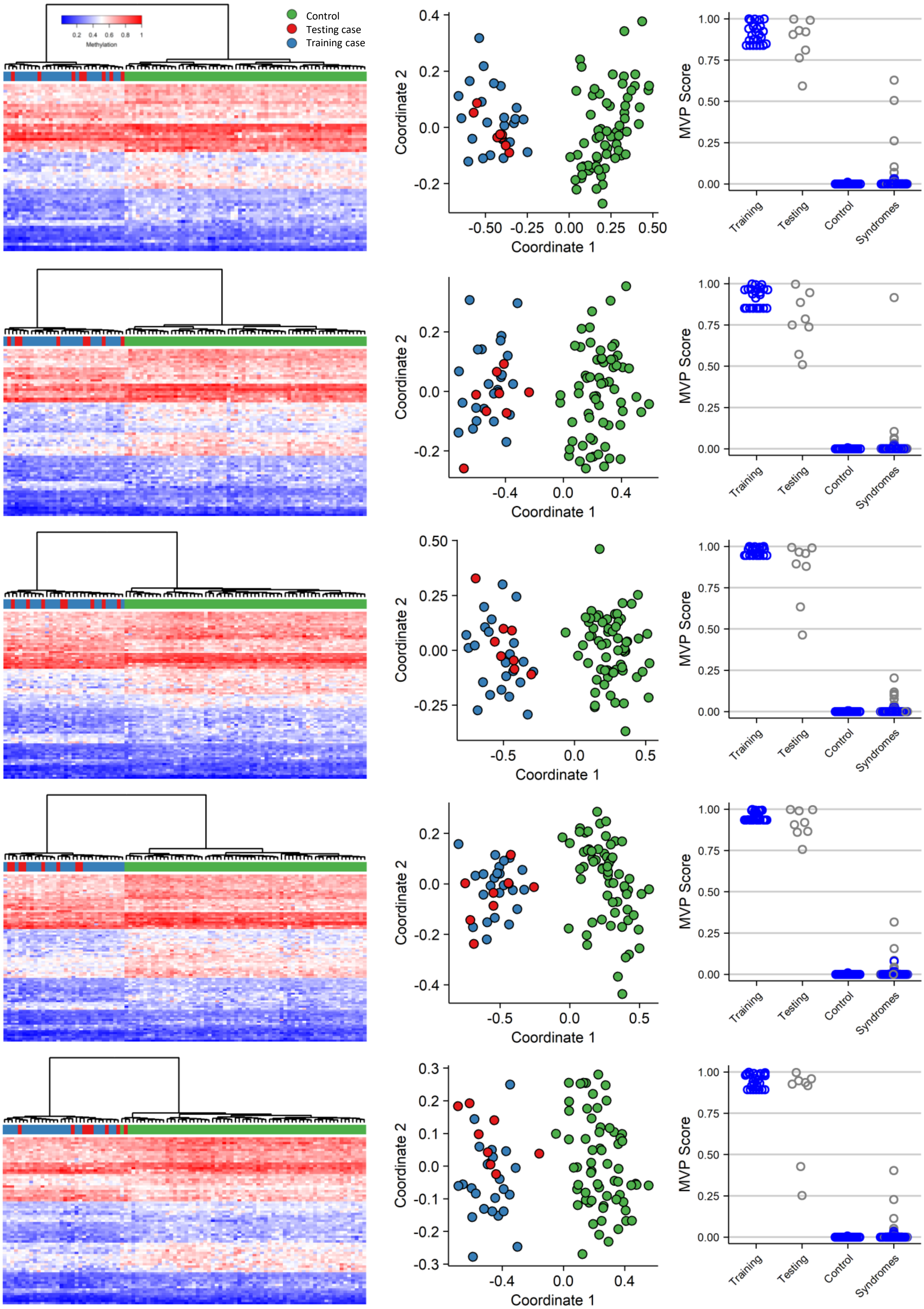

Supplementary Figure 4: Leave-25%-out cross validation results

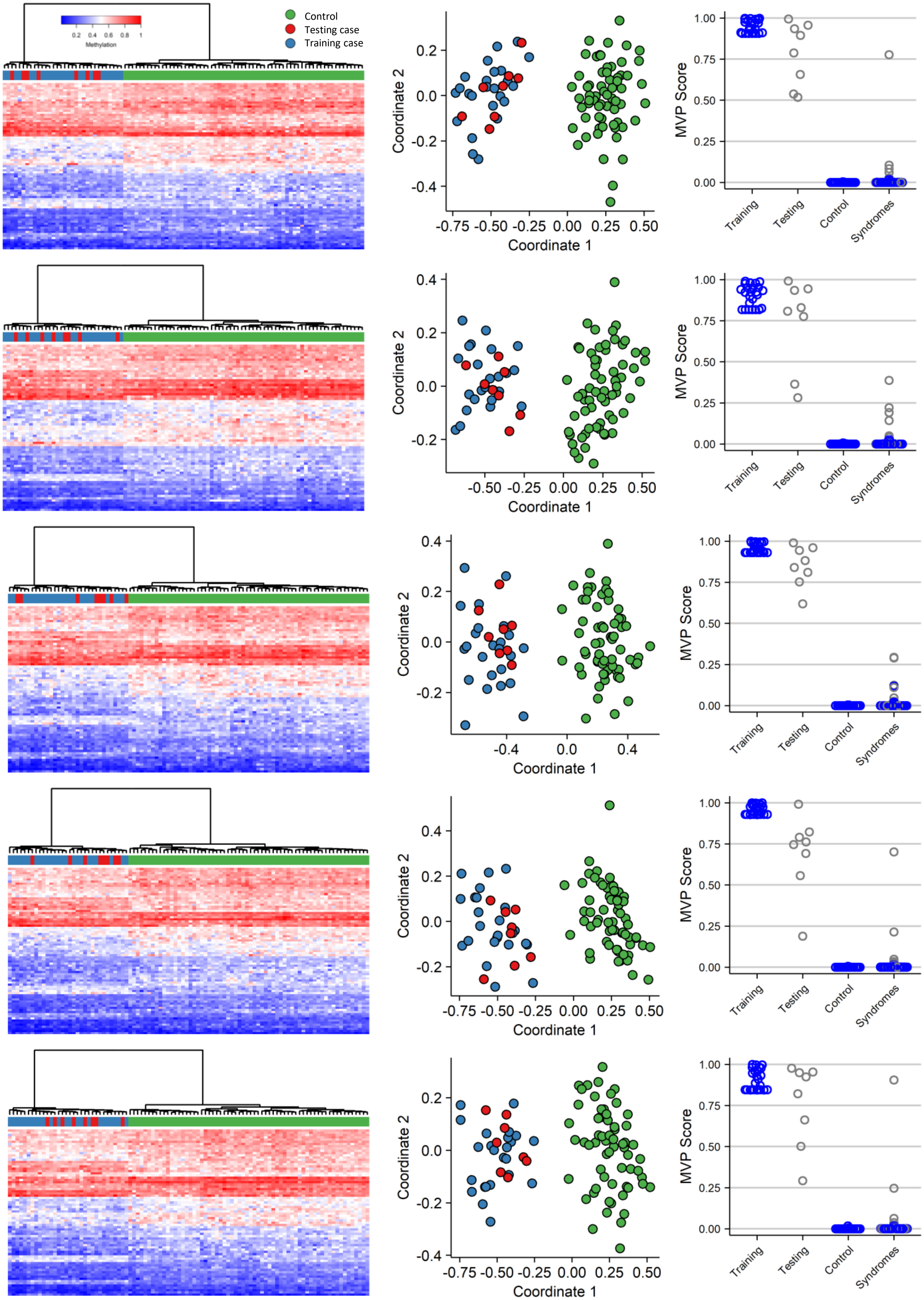

Supplement: Supplementary file 1 [file ijms-22-08611-s001.zip › ijms-1311593-sup/Supplementary_images_3_4.pdf]
